# Surveillance de la rougeole en Côte d’Ivoire de 2010 à 2022: Épidémiologie – Facteurs associés – Recommandations

**DOI:** 10.4102/jphia.v17i1.1743

**Published:** 2026-04-16

**Authors:** Kouadio D. Ekra, Calixte H.H. Guehi, Guillaume Okoubo, Éric M. Ahoussou

**Affiliations:** 1 Département de Santé Publique, Université Félix Houphouët Boigny, UFR des Sciences Médicales, Abidjan, Côte d’Ivoire; 2 Institut National d’Hygiène Publique, Abidjan, Côte d’Ivoire

**Keywords:** measles, epidemiology, associated factors, COVID-19, Côte d’Ivoire

## Abstract

**Measles surveillance in Côte d’Ivoire from 2010 to 2022: Epidemiology – Associated factors – recommendations**.

**Background:**

In 2011, African countries planned to eliminate measles by 2020. Since this goal was not achieved, what was the epidemiological profile of measles in Côte d’Ivoire from 2010 to 2022?

**Aim:**

To assess the epidemiological situation of measles in Côte d’Ivoire from 2010 to 2022.

**Setting:**

Côte d’Ivoire, a country in West Africa.

**Methods:**

Retrospective cross-sectional study of measles surveillance data from 2010 to 2022. These data were obtained from health district reports and biological diagnosis by the l’Institut Pasteur de Côte d’Ivoire (IPCI). We analysed the data and created maps using R4.2.1 and ArcGIS 10.7 software.

**Results:**

A total of 6584 (24.3%) confirmed cases were reported out of 27 090 notifications between 2010 and 2022. The annual incidence increased steadily from 23 to 53 cases per 1 000 000 inhabitants between 2018 and 2022, with a peak observed in 2021 (67 cases per 1 000 000 inhabitants). Children aged 0–5 years accounted for 67% of cases; 81% of cases were unvaccinated. Multivariate analysis noted that the risk of measles was higher among children aged 0–5 years, in rural areas (adjusted odds ratio [AOR] 1.26, *p* = 0.001), during COVID-19 (AOR 1.90 [95% confidence interval [CI] 1.80–2.02], *p* = 0.001), and in the absence of measles vaccination.

**Conclusion:**

These measures against COVID-19 have resulted in a decrease in public demand for preventive health services such as vaccination due to fear of contracting COVID-19 in healthcare facilities.

**Contribution:**

Guiding the fight against measles through innovative recommendations.

## Introduction

La rougeole est une maladie infectieuse extrêmement contagieuse causée par un virus du genre *Morbillivirus*. Elle est transmise par voie aérienne. Certaines complications sont potentiellement mortelles (pneumonie, encéphalite).

Avant l’introduction de la vaccination contre la rougeole en 1963 et sa généralisation, on enregistrait tous les deux ou trois ans d’importantes épidémies qui pouvaient causer environ 2.6 millions de décès chaque année^1^. Au regard des succès du programme de vaccination contre la variole, la Vingt-Septième Assemblée Mondiale de la Santé a préconisé le 23 mai 1974 ‘d’élargir’ la vaccination contre la variole à six maladies cibles meurtrières dans la petite enfance et disposant d’un vaccin à savoir la tuberculose, la poliomyélite, le tétanos, la diphtérie, la coqueluche et la rougeole^2^ ; marquant ainsi l’acte de naissance du ‘Programme Élargi de Vaccination’.

La mise en œuvre de cette recommandation associée à différentes stratégies, a permis une réduction des décès imputables à la rougeole dans le monde de 78%, passant de 733 000 en 2000 à 164 000 en 2008^3^.

L’élimination de la rougeole paraissant possible, les diverses régions de l’Organisation Mondiale de la Santé (OMS) ont mis en place des stratégies pour y parvenir. Ainsi, en 2011, les pays africains de la région Afrique de l’OMS prévoyaient l’élimination de la rougeole à l’horizon 2020^4^. La stratégie régionale d’élimination de la rougeole visait à atteindre une incidence de la rougeole inférieure à 1 cas pour 1 000 000 d’habitants au niveau national ; une couverture vaccinale contre la rougeole d’au moins 95% au niveau national et dans tous les districts. En 2021, la 71ème session du comité régional Afrique concluait à une non atteinte de l’objectif régional d’élimination^5^. En effet, en 2019, la couverture vaccinale pour la 1ère dose de rougeole (CV_RR1) était de 69% contre 71% en 2011 dans la région africaine. Cette couverture était de 33% pour la deuxième dose du vaccin antirougeoleux (CV_RR2), selon les estimations de l’OMS et de l’UNICEF [United Nations of International Children’s Emergency Fund]^6^.

En 2020, l’OMS et ses partenaires ont défini un nouveau cadre stratégique de lutte contre la rougeole et la rubéole dont le but était d’atteindre de façon durable les objectifs régionaux d’élimination de la rougeole et de la rubéole en 2030^7^.

Devant cette non-atteinte régionale des objectifs de l’élimination de la rougeole, il convient de comprendre la situation épidémiologique des pays en vue d’ajuster les stratégies de lutte. Ainsi, cette étude vise à évaluer la situation épidémiologique de la rougeole en Côte d’Ivoire de 2010–2022, plus spécifiquement à analyser des tendances temporo-spatiales de la rougeole en Côte d’Ivoire de 2010 à 2022 et à identifier les facteurs associés à la survenue de la rougeole de 2010 à 2022.

## Méthode

### Cadre de l’étude

La Côte d’Ivoire est située en Afrique de l’Ouest, dans la zone intertropicale, sur le golfe de Guinée. Elle couvre une superficie de 322 462 km^2^. Le pays est bordé au sud par l’océan Atlantique, à l’Est par le Ghana (640 km), au Nord par le Burkina Faso (490 km) et le Mali (370 km) et à l’Ouest par la Guinée (610 km) et le Liberia (580 km). Sa population est estimée à 29 389 150 habitants selon le Recensement Général de la population et de l’Habitat (RGPH) en 2021^8^.

### Définition des cas de rougeole^9^

#### Cas suspect

Toute personne présentant de la fièvre, une éruption maculopapuleuse (non vésiculaire) généralisée et de la toux, un coryza ou une conjonctivite ou toute personne chez qui le clinicien suspecte la rougeole.

#### Cas confirmé

Cas suspect confirmé par le laboratoire (anticorps IgM [immunoglobuline M] positif) ou lien épidémiologique à des cas confirmés lors d’une épidémie.

### Notification

Devant la présence de signes faisant penser à la rougeole (fièvre, éruption généralisée maculopapuleuse (non vésiculaire) et toux, rhume ou conjonctivite), le centre de santé ou l’hôpital notifiait ce cas suspect au district sanitaire à l’aide de formulaire de notification et faisait aussi un prélèvement de sérum sanguin qui était acheminé à l’Institut Pasteur à Abidjan pour le diagnostic biologique. Le chargé de surveillance du district se chargeait de saisir le formulaire de notification dans la base de données anciennement Episurveyor (MAGPI) et envoyait le formulaire scanné à la Direction de Coordination du Programme Élargi de Vaccination (DC-PEV). Des enquêtes étaient menées pour identifier des contacts et évaluer l’étendue de la flambée.

#### Collecte et transport des échantillons

Selon le Guide de Surveillance Intégrée des maladies et Riposte (SIMR)^9^, l’établissement de santé faisait le prélèvement du sérum dès la première consultation pour le diagnostic (la présence dans le sérum d’anticorps Immunoglobuline M (IgM), contre le virus de la rougeole). Les directives sur la manière de prélever le sérum, de le conserver et de l’expédier, le moment où les résultats devraient être disponibles, les sources et compléments d’information, sont mentionnées dans ledit Guide.

#### Diagnostic biologique

Le diagnostic biologique pour la confirmation du diagnostic était fait à l’Institut Pasteur de Côte d’Ivoire (IPCI). La recherche de la présence dans le sérum d’anticorps IgM, contre le virus de la rougeole permettait de confirmer le diagnostic.

#### Définitions opérationnelles

**Reconstitution des districts sanitaires:** En 2019, l’arrêté *087/MSHP/CAB du 08 Mai 2019* portant organisation et composition des régions sanitaires du Ministère de la santé, a permis de passer de 86 à 113 districts sanitaires. Nous avons considéré pour cette analyse l’existence des 113 districts depuis 2010. Pour cela, nous avons reconstitué les districts sanitaires en nous basant sur les centres de santé qui n’ont pas changé.

**Incidence élevée:** Incidence égale ou supérieure à 100 cas pour 1 000 000 d’habitants.

### Type d’étude

Il s’agissait d’une étude transversale rétrospective à visée analytique portant sur les données de surveillance de la rougeole et de la vaccination antirougeoleuse du Programme Élargi de Vaccination de 2010–2022 (données administratives c’est-à-dire les données de vaccination déclarées par le pays). Nous avons également utilisé les données de couverture vaccinale estimée de l’OMS et de l’UNICEF^6^. Ces données proviennent des notifications des districts sanitaires et du diagnostic biologique de l’Institut Pasteur de Côte d’Ivoire^1^.

La couverture vaccinale WUENIC (WHO [*World Health Organization*]/UNICEF [*Estimates of National Immunization Coverage*] est estimée à partir des données de couverture vaccinale administrative, d’enquête de couverture vaccinale et des données de surveillance.

### Période d’étude

Les données analysées ont été recueillies sur la période de 2010 à 2022 (de la 1^ère^ à la 52^ème^ semaine épidémiologique pour chaque année).

### Population d’étude

Il s’agit des cas notifiés de rougeole.

### Variables étudiées

Les variables étudiées étaient les suivantes: âge, sexe, district sanitaire de notification, milieu de résidence, période COVID-19, nombre de dose de vaccin antirougeoleux reçu, hospitalisation, issue de la maladie, classification finale, couvertures vaccinales annuelles contre la rougeole.

Pour ces différentes variables d’intérêt, il n’y avait pas de données manquantes en dehors de l’âge, qui comptait 17 données manquantes soit 0.06% (17/27 090).

### Méthodes statistiques

Le test du khi-deux d’indépendance, le test de Wilcoxon-Mann-Whitney et le test exact de Fisher ont été utilisés pour comparer les variables qualitatives et quantitatives selon la classification finale des cas suspects (cas confirmés ou cas non confirmés/négatifs).

A l’aide d’une régression logistique univariée, nous avons recherché les associations entre la survenue de rougeole et les variables suivantes: âge, sexe, milieu de résidence, période covid-19, nombre de dose de vaccin antirougeoleux reçu, hospitalisation, issue de la maladie. Les variables qui étaient associées au seuil de 25% ont été retenues dans le modèle multivarié initial. Nous avons ensuite fait une régression logistique pas à pas descendante à l’issue de laquelle les variables significatives au seuil de *p* < 5% ont été retenues dans le modèle final.

Les analyses ont été effectuées à l’aide du logiciel R version 4.2.1.

Le logiciel ArcGIS 10.7 a été utilisé pour réaliser les représentations cartographiques.

### Considérations éthiques

L’autorisation éthique (Ref: 003-2025/MSHP/CEIRSVS-INHP) pour mener cette étude a été obtenue auprès du comité d’éthique institutionnel de la recherche en sciences de vie et de la santé de l’Institut National d’Hygiène Publique. Les données ont été fournies par la Direction de la coordination du Programme élargi de vaccination en Côte d’Ivoire. Toutes les informations identifiables ont été supprimées avant l’analyse. L’ensemble de données a été stocké de manière sécurisée sous forme de fichier protégé par mot de passe à l’aide de Microsoft^®^ Excel 2016.

## Résultats

### Répartition temporelle des cas de rougeole

De 2010 à 2022, 27 090 cas suspects de rougeole ont été notifiés par les établissements de santé à la DC-PEV via les districts sanitaires. Un total de 6584 cas confirmés de rougeole a été diagnostiqué soit 24.3% des cas suspects sur les 13 années de surveillance. Dans l’ensemble, on peut noter que les cas confirmés de rougeole augmentent avec les notifications de cas suspects (Figure 1). Les campagnes de vaccination ont eu un impact faible sur l’incidence de la rougeole. En effet, l’incidence est passée de 18 à 14/1 000 000 de 2018 à 2019 soit une réduction de 22% et de 67 en 2021 à 53 en 2022 soit une réduction de 20%.

**FIGURE 1:**
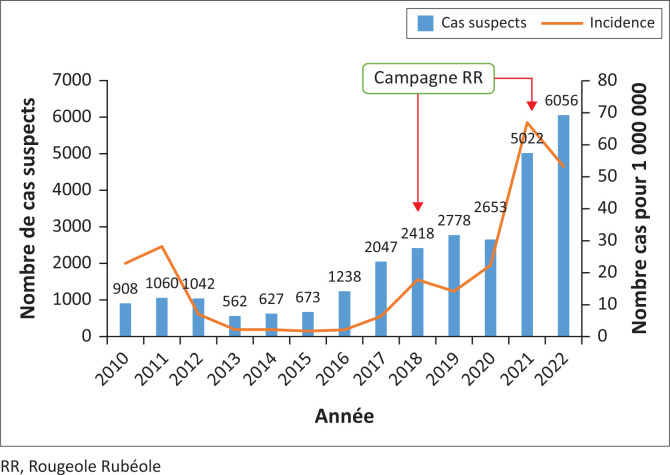
Évolution des notifications des cas suspects et de l’incidence de la rougeole en Côte d’Ivoire, 2010–2022 (*N* = 6584).

De 2010–2022, l’évolution de l’incidence de la rougeole peut être scindée en trois phases (Figure 2):

- Une 1^ère^ phase de diminution importante de l’incidence allant de 2010 à 2016. Pendant cette phase, l’incidence de la rougeole est passée de 23 cas /1 000 000 habitants en 2010 à 2 cas en 2016 soit une diminution de l’incidence de 91.3%. Pendant cette phase, on note des efforts d’amélioration de la couverture vaccinale estimée pour la 1^ère^ dose de rougeole passant de 49% à 73%.
- La 2^ème^ phase va de 2017 à 2019. Elle est marquée par une augmentation modérée de l’incidence de la rougeole. En effet, le taux d’incidence pour 1 million d’habitants est passé de 6 en 2017 à 14 en 2019 soit une hausse de 133%. On observe le maintien de la CV_RR1 estimée à une moyenne de 72%.
- La 3^ème^ phase allant de 2020 à 2022 est marquée par une augmentation importante de l’incidence de la rougeole qui est passée de 22 en 2020 à 53 en 2022 (avec un pic de 67 en 2021) soit une hausse de 141%. On constate une baisse de la CV_RR1 pendant cette phase passant de 63% à 62%.

**FIGURE 2:**
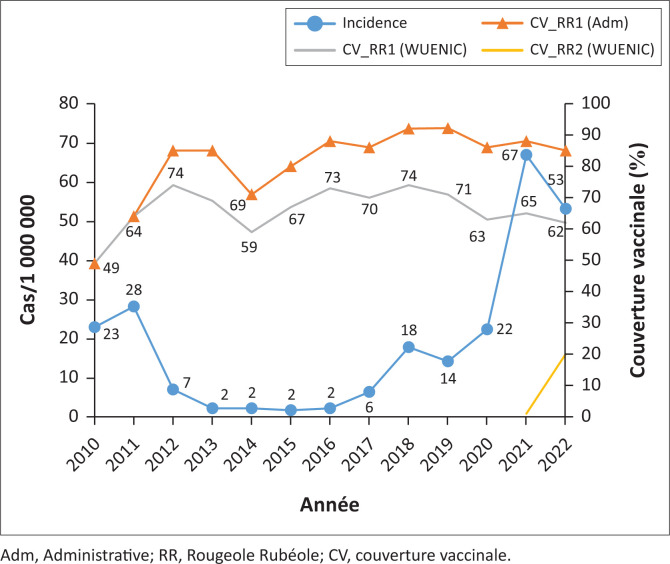
Couverture vaccinale antirougeoleuse (CV_RR)^6^ (administrative et estimée OMS/UNICEF) et évolution de l’incidence de la rougeole, Côte d’Ivoire, 2010–2022.

La courbe épidémiologique mensuelle (Annexe 1, Figure 1-A1), montre une tendance saisonnière avec un maximum de cas de rougeole dans les 6 premiers mois de chaque année.

### Répartition spatiale des notifications des cas suspects, des cas confirmés et de la couverture vaccinale administrative

Le nombre de districts sanitaires à incidence élevée (égale ou supérieure à 100 cas/1 000 000 habitants) a augmenté régulièrement de 2018 à 2022. Ces districts sont passés ainsi de 5 à 17 avec un pic de 23 districts en 2021. Les districts sanitaires qui avaient au moins une incidence annuelle élevée de rougeole sur cette période étaient de 41. Parmi ceux-ci, 11 avaient au moins deux incidences annuelles élevées de rougeole (Annexe 1, Figure 3-A1).

La Figure 3 révèle les relations existant entre la couverture vaccinale, la qualité de la surveillance (notification des cas suspects) et l’incidence de la rougeole. En effet, lorsque la couverture vaccinale n’est pas bonne et la notification de cas suspects est optimale (au moins 100 notifications/1 000 000 habitants), l’incidence de la maladie semble aussi élevée. Cependant, même dans les districts où la CV_RR1 administrative est bonne (égale ou supérieure à 95%) lorsque les notifications de cas suspects sont optimales, on peut observer une incidence élevée de rougeole. Les analyses complémentaires (Annexe 1, Tableau 1-A1) ont montré que dans une telle situation, la plupart des cas étaient plus âgés (âge égale ou supérieur à 5 ans) que dans les districts où la couverture vaccinale n’était pas bonne (CV_RR1 < 95%).

**FIGURE 3:**
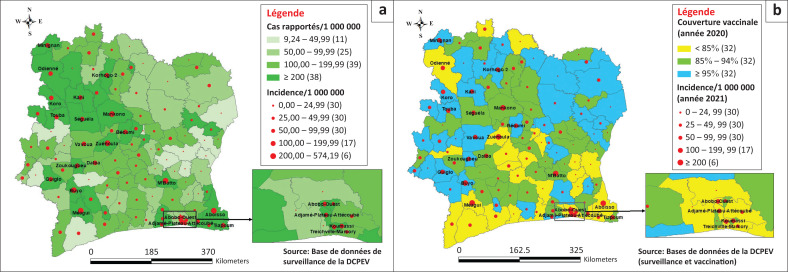
Répartition spatiale de l’incidence de la rougeole en 2021 selon la notification des cas suspects et la couverture vaccinale administrative antirougeoleuse de 2020 dans les districts sanitaires, Côte d’Ivoire. (a) Nombre de cas suspects rapportés/1 000 000 habitants et incidence de la rougeole par district sanitaire en 2021 (*N* = 1966). (b) Couverture vaccinale en RR de 2020 et incidence de la rougeole par district sanitaire en 2021 (*N* = 1966).

L’analyse spatiale note également, l’existence d’une relation entre and non en la densité de la population et l’incidence de la rougeole dans le sens d’une augmentation de l’incidence quand la densité est forte (Annexe 1 - Figure 2-A1).

### Caractéristiques des cas confirmés et des cas non confirmés

Comparés aux cas non confirmés de rougeole, les cas confirmés (Tableau 1) étaient moins jeunes (âge médian 3 ans vs 4 ans; intervalle interquartile [IIQ]: 1–7; *p* = 0.001), résidaient plus en milieu rural (53.3 % vs 47.8%, *p* = 0.001), n’avaient le plus souvent pas reçu de dose de vaccin contre la rougeole (81.3% vs 65.4%, *p* = 0.001), étaient le plus hospitalisés (1.3% vs 0.8%; *p* = 0.001). Les cas confirmés étaient plus fréquents en période de COVID-19 qu’avant cette période (63.4% vs 36.6%; *p* = 0.001).

**TABLEAU 1:** Analyse comparative des caractéristiques des cas confirmés et des négatifs, Côte d’Ivoire, 2010–2022 (*N* = 27 090).

Caractéristiques	Cas négatif (*n* = 20 506)				Cas confirmé (*n* = 6584)				Total				*p*-value
	*n*	%	Médiane	IIQ	*n*	%	Médiane	IIQ	*n*	%	Médiane	IIQ	
**Age (année)**	-	-	4	1–8	-	-	3	1–7	-	-	4	1–8	< 0.001
Manquant	-	17.0	-	-	-	0.0	-	-	-	17.0	-	-	-
**Groupe d’âge (année)**	-	-	-	-	-	-	-	-	-	-	-	-	< 0.001
≥ 15	2183	107.0	-	-	700	10.6	-	-	2883	10.6	-	-	-
5–14	5494	26.8	-	-	1494	22.7	-	-	6988	25.8	-	-	-
0–5	12 812	62.5	-	-	4390	66.7	-	-	17 202	63.5	-	-	-
Manquant	-	17.0	-	-	-	0.0	-	-	-	17.0	-	-	-
**Sexe**	-	-	-	-	-	-	-	-	-	-	-	-	0.500
Féminin	9545	46.5	-	-	3095	47.0	-	-	12 640	46.7	-	-	-
Masculin	10 961	53.5	-	-	3489	53.0	-	-	14 450	53.3	-	-	-
**Période COVID-19**	-	-	-	-	-	-	-	-			-	-	< 0.001
Avant 2020	10 951	53.4	-	-	2408	36.6	-	-	13 359	49.3	-	-	-
Après 2020	9555	46.6	-	-	4176	63.4	-	-	13 731	50.7	-	-	-
**Issue**	-	-	-	-	-	-	-	-	-	-	-	-	0.400
Guérison	20 478	99.9	-	-	6578	99.9	-	-	27 056	99.9	-	-	-
Décès	28	0.1	-	-	6	0.1	-	-	34	0.1	-	-	-
**Milieu de résidence**	-	-	-	-	-	-	-	-	-	-	-	-	< 0.001
Urbain	10 711	52.2	-	-	3074	46.7	-	-	13 785	50.9	-	-	-
Rural	9795	47.8	-	-	3510	53.3	-	-	13 305	49.1	-	-	-
**Dose de vaccin RR reçu**	-	-	-	-	-	-	-	-	-	-	-	-	< 0.001
1 dose de vaccin	6122	29.9	-	-	1069	16.2	-	-	7191	26.5	-	-	-
2 doses et plus	972	4.7	-	-	161	2.4	-	-	1133	4.2	-	-	-
Aucune dose de vaccin	13 412	65.4	-	-	5354	81.3	-	-	18 766	69.3	-	-	-
**Hospitalisation**	-	-	-	-	-	-	-	-	-	-	-	-	< 0.001
Non	20 337	99.2	-	-	6498	98.7	-	-	26 835	99.1	-	-	-
Oui	169	0.8	-	-	86	1.3	-	-	255	0.9	-	-	-

Note: *n* (%); Médiane (EI). Test du khi-deux d’indépendance; test de Wilcoxon-Mann-Whitney; test exact de Fisher.

RR, Rougeole Rubéole; IIQ, intervalle interquartile.

### Facteurs associés à la rougeole

Dans l’analyse multivariée (Tableau 2), on observe une association significative entre la rougeole et les facteurs suivants: le groupe d’âge ([0–5 ans] contre ≥ 15 ans Rapport de Cotes ajusté [RCa] 1.33 [95% Intervalle de Confiance {IC} 1.21–1.46]); ([5–14 ans] contre ≥ 15 ans RCa 1.03 [95% IC 0.93–1.14], *p* = 0.001), la résidence en milieu rural (RCa 1.26 [95% IC 1.19–1.34], *p* = 0.001), la période de la COVID-19 (RCa 1.90 [95% IC 1.80–2.02], *p* = 0.001), n’avoir reçu aucune dose de vaccin antirougeoleuse (Aucune dose de vaccin contre 1 dose de vaccin (RCa 2.36 [95% IC 2.19 2.54] *p* = 0.001); 2 doses et plus contre 1 dose de vaccin (RCa 0.99 [95% IC 0.82–1.18]), hospitalisation (RCa 1.93 [95% IC 1.47–2.52], *p* = 0.001).

**TABLEAU 2:** Association entre les caractéristiques et la rougeole, Côte d’Ivoire, 2010–2022 (*N* = 27 090).

Variables	Analyse univariée			Analyse multivariée		
	RC	IC_95%_	*p*-value	RCa	IC_95%_	*p*-value
**Groupe d’âge (année)**	-	-	< 0.001	-	-	< 0.001
≥ 15	1.00	-	-	1.00	-	-
5-14	0.85	0.77–0.94	-	1.03	0.93–1.14	-
0–5	1.07	0.98–1.17	-	1.33	1.21–1.46	-
**Sexe**	-	-	0.500	-	-	-
Féminin	1.00	-	-	-	-	-
Masculin	0.98	0.93–1.04	-	-	-	-
**Milieu de résidence**	-	-	< 0.001	-	-	< 0.001
Urbain	1.00	-	-	1.00	-	-
Rural	1.25	1.18–1.32	-	1.26	1.19–1.34	-
**Période COVID-19**	-	-	< 0.001	-	-	< 0.001
2010–2019	1.00	-	-	1.00	-	-
2020–2022	1.99	1.88–2.10	-	1.90	1.80–2.02	-
**Dose de vaccin RR reçu**	-	-	< 0.001	-	-	< 0.001
1 dose vaccin	1.00	-	-	1.00	-	-
2 doses et plus	0.95	0.79–1.13	-	0.99	0.82–1.18	-
Aucune dose de vaccin	2.29	2.13–2.46	-	2.36	2.19–2.54	-
**Hospitalisation**	-	-	< 0.001	-	-	< 0.001
Non	1.00	-	-	1.00	-	-
Oui	1.59	1.22–2.06	-	1.93	1.47–2.52	-
**Issue**	-	-	0.300	-	-	-
Guérison	1.00	-	-	-	-	-
Décès	0.67	0.25–1.50	-	-	-	-

RC, Rapport de Cotes; IC, Intervalle de Confiance; RCa, Rapport de Cotes ajusté.

## Discussion

La rougeole est classée parmi les maladies à potentiel épidémique qui nécessitent une notification immédiate. Cette maladie évitable par la vaccination continue encore de toucher les populations et attire l’intérêt des politiques de santé publique.

Dans notre étude, la courbe d’évolution de l’incidence de la rougeole est similaire à celle décrite par d’autres auteurs. En effet, selon l’OMS^10^ et le CDC,^11^ l’incidence annuelle de la rougeole a diminué de 88% à l’échelle mondiale entre 2000 et 2016, puis a augmenté entre 2017 et 2019. Cependant, en Côte d’Ivoire cette phase de diminution de l’incidence semble être liée à une insuffisance dans les indicateurs de surveillance au regard des faibles notifications des cas suspects observés entre 2013 et 2015 alors qu’à cette même période, les couvertures vaccinales estimées étaient inférieures à 70%.

**TABLEAU 3:** Propositions de critères de classification des districts sanitaires et des interventions.

Zones de priorité	Critères	Interventions
Risque élevé	Au moins 2 incidences annuelles élevées de rougeole ou Densité de population élevée (1000 hbts/km^2^)	Seuil de notification de cas suspects (au moins 100/1 million d’hbts) Dénombrement Faire des AVS en s’aidant du GTS LQAS/supervision trimestrielle
Risque moyen	1 incidence annuelle élevée de rougeole ou Densité de population moyenne (100 hbts/km^2^ – 999 hbts/km^2^)	Seuil de notification de cas suspects (au moins 100/ 1 million d’hbts) LQAS/supervision semestrielle
Risque faible	Incidence annuelle de rougeole pas élevée ou Densité de population faible (< 100 hbts/km^2^)	Seuil de notification de cas suspects (au moins 100/1 million d’hbts) LQAS/supervision annuelle

Hbts, habitants; LQAS, lot quality assurance sampling; AVS, activités de vaccination supplémentaires.

La hausse de l’incidence entre 2017 et 2019 est en lien avec l’amélioration des notifications des cas suspects Cette amélioration des notifications est probablement liée à l’amélioration du système de surveillance par l’effet conjugué des formations des épidémiologistes de terrain, le déploiement de consultants dans les districts, les réunions hebdomadaires de suivi avec les districts et l’accroissement du nombre de districts sanitaires en 2019, toujours dans un contexte de couverture vaccinale estimée non optimale.

Les campagnes de vaccination semblent avoir un effet limité, selon les données, sur les flambées de rougeole Nous ne pouvons cependant pas être affirmatifs en dehors d’une étude qui adresserait spécifiquement cette question.

Notre étude note que la période de la COVID-19 est significativement associée à une augmentation de l’incidence de la rougeole, comme on l’observe dans la dernière phase (2020–2022) de la courbe de l’incidence; certainement en rapport avec les effets négatifs des décisions prises pendant la pandémie de COVID-19. En effet, après la déclaration de la pandémie de COVID-19, plusieurs mesures ont été prises par les pays pour contenir la propagation du virus et atténuer ses effets. On peut citer des mesures telles que l’interdiction de rassemblement publique, le confinement, les interdictions de voyager, la quarantaine, la distanciation sociale et la réaffectation des agents de santé à la réponse contre la COVID-19. Ces mesures ont eu pour conséquence la diminution de la demande et de l’offre publique de services de santé préventifs comme la vaccination^12^ en raison de la peur de contracter la COVID-19 dans les établissements de soins. Quant à la surveillance de la rougeole, on a observé une faible notification des cas suspects en 2020 comparé aux années antérieures allant jusqu’à 50% dans certains pays^13^, une réduction de la sollicitation des services de vaccination de routine donc une baisse de la couverture vaccinale. La sous vaccination des cibles a été responsable des flambées épidémiques donc de l’augmentation de l’incidence de la rougeole observée en 2021 et 2022. Pendant la pandémie de COVID-19, de nombreux pays dans le monde, comme en Afrique, ont connu une baisse de la couverture vaccinale et des performances de surveillance. Dans le monde, la couverture vaccinale infantile a diminué pour la plupart des vaccins en raison de la pandémie de la COVID-19 en 2021, avec la première dose du vaccin contre la rougeole, celle-ci qui a diminué à 81%, soit le niveau le plus bas depuis 2008^14^. Avant la Covid-19, sur une période de 5 ans (2014–2018), dans la région Africaine de l’OMS, 6710 cas de rougeole confirmés en laboratoire ont été dénombrés contre 11 781 cas pour la seule année 2020^13^.

Les enfants de moins de 5 ans sont plus à risque de contracter la rougeole que les plus âgés. C’est la tranche d’âge la plus touchée, ce qui explique d’ailleurs qu’elle soit la cible de la vaccination de routine et des activités de vaccination supplémentaires. Cependant, 33.3% des personnes affectées par la rougeole sont plus âgées donc susceptibles de transmettre la rougeole à la cible actuelle de la vaccination^15^. Dans la perspective de l’élimination de la rougeole, qui ne fait d’ailleurs pas mention de l’âge^4^, il importe d’adapter les cibles de la vaccination afin de prendre en compte les personnes non immunisées et/ou à risque.

Notre étude réaffirme l’intérêt de se faire vacciner contre la rougeole. En effet, ceux qui n’ont reçu aucune dose de vaccin sont plus à risque de faire la rougeole que ceux qui n’ont reçu qu’ une seule dose. Aussi l’intérêt de la deuxième dose qui protège mieux qu’une seule dose est mis en évidence. D’autres études^14,16^ ont noté que les enfants non vaccinés étaient plus à risque de faire la rougeole 2.63 (1.99–3.47); *p* = 0.001. Ces études ont également montré le caractère protecteur d’au moins deux doses de vaccin antirougeoleux 0.39 (0.23–0.68); *p* = 0.002. Le taux de protection contre la rougeole augmente jusqu’à 99 % après la deuxième dose de vaccin^17,18,19,20,21,22,23^ et le vaccin procure une immunité à long terme après administration de deux doses^24,25^.

Les personnes résidant en milieu rurale sont plus susceptibles d’avoir la rougeole probablement du fait de certains facteurs comme l’accessibilité aux centres de santé, le faible niveau de revenu et le niveau de littératie en milieu rurale^26^. Les résultats d’une méta-analyse en Éthiopie notaient que les enfants nés de mères illettrées avaient 1.4 fois plus de risque de contracter la rougeole^16^.

Enfin, nous avons aussi montré qu’avoir la rougeole accroit la probabilité d’être hospitalisé et donc augmente les dépenses de santé des familles. Il s’agit d’un argument de poids pour sensibiliser les familles à bien faire vacciner leurs enfants.

### Limites

Cette étude présente quelques limites inhérentes à son caractère rétrospectif et à l’utilisation des données de surveillance de la rougeole. En effet, les résultats reposent sur les cas notifiés dans les structures de santé, ce qui peut exposer à une sous-estimation de l’incidence réelle, en particulier pour les cas bénins ou non pris en charge médicalement. La sensibilité du système de surveillance a pu varier selon les performances du système de surveillance dans les districts.

La qualité des données pourrait aussi constituer une limite importante. Cependant, sur les variables clés de notre étude, seule l’âge avait quelques données manquantes (17 sur un total de 27 090 cas suspect) et ce parmi les cas non confirmés de rougeole.

En dépit de ces limites, les résultats de ce travail apportent des informations essentielles sur l’évolution spatio-temporelle de la rougeole en Côte d’Ivoire et constituent une base solide pour orienter les stratégies de surveillance et de prévention.

### Recommandations/stratégies

Le calendrier vaccinal (routine et/ou rattrapage) doit être modifié pour être adapté aux données épidémiologiques et dans l’objectif d’élimination de la rougeole et surtout parce que la cible actuelle dans ses interactions avec les autres n’est pas épargnée des risques de contamination. Il faut étendre la vaccination aux personnes âgées de plus de 5 ans qui représentent 33% des cas de rougeole car ceux-ci pourraient transmettre la maladie aux enfants de moins de 5 ans non vaccinés ou sous-vaccinés. Les pays qui sont parvenus à éliminer la rougeole ont vacciné des populations plus âgées En effet, aux USA and non US, à l’occasion de l’introduction d’une deuxième dose du vaccin contre la rougeole en 1989, le Comité consultatif des pratiques de vaccination a recommandé que les enfants d’âge scolaire ainsi que les jeunes adultes fréquentant l’université reçoivent une deuxième dose de vaccin contre la rougeole^27,28^. À Cuba, en 1986, une campagne de masse a été faite chez les enfants de 1 an à 14 ans entrainant une baisse rapide et importante de l’incidence de la maladie et conduisant le pays vers l’élimination^28^. Le Groupe stratégique consultatif d’experts sur la vaccination (SAGE) a recommandé, au regard des résultats de modélisation et des enquêtes sérologiques, pour parvenir à l’élimination de la rougeole, une couverture d’au moins 95% de chaque cohorte de naissance avec 2 doses du vaccin contre la rougeole dans chaque pays dans les programmes de vaccination systématiques. Les pays devront aussi s’efforcer d’identifier les tranches d’âge et les sous-groupes de population particuliers présentant des lacunes immunitaires, c’est-à-dire dont le taux d’immunité est inférieur à 95%, et proposer des activités de vaccination de rattrapage en conséquence^29^. Dans la région Africaine de l’OMS, des campagnes de vaccination supplémentaires, ciblant les enfants de 9 mois à 14 ans, ont permis la baisse moyenne du nombre de cas de rougeole déclarés de 91% sur les 2 années suivant lesdites campagnes^30^.

La vaccination contre la rougeole pourrait être proposée donc aux groupes à risque qui seraient identifiés comme les voyageurs, les professionnels de santé, les personnes âgées de plus de 5 ans sans antécédents de rougeole et qui n’ont jamais été vaccinées.

Il faudrait associer aux efforts actuels, une meilleure organisation des campagnes de vaccination pour avoir un impact immédiat et durable sur les flambées de rougeole (paiement accéléré des acteurs, communication, lutte contre les rumeurs, Geo-tracking system [GTS] dans les districts à incidence élevée de rougeole). L’allure de la courbe épidémiologique suggère qu’il faudrait faire les activités de vaccination supplémentaire vers les deux derniers mois de l’année afin de réduire les flambées observées les 6 premiers de l’année.

Adopter une nouvelle stratégie de surveillance en classant les districts en zone de priorités sur la base des critères suivants: historique épidémiologique du district, densité de population.

Ainsi un ensemble d’intervention serait attribué selon la zone de priorité du district.

## Conclusion

L’évolution annuelle de l’incidence de la rougeole en Côte d’Ivoire de 2010–2022 répète à s’y méprendre celle du niveau mondial avec l’impact négatif de la pandémie de la COVID-19 de 2020–2022. Des mesures comme le Grand Rattrapage sont en cours actuellement en Côte d’Ivoire en vue d’offrir les services de vaccination aux enfants de 2 à 5 ans sous vaccinés ou non vaccinés du fait de la pandémie à coronavirus.

## Figures and Tables

**FIGURE 1-A1:**
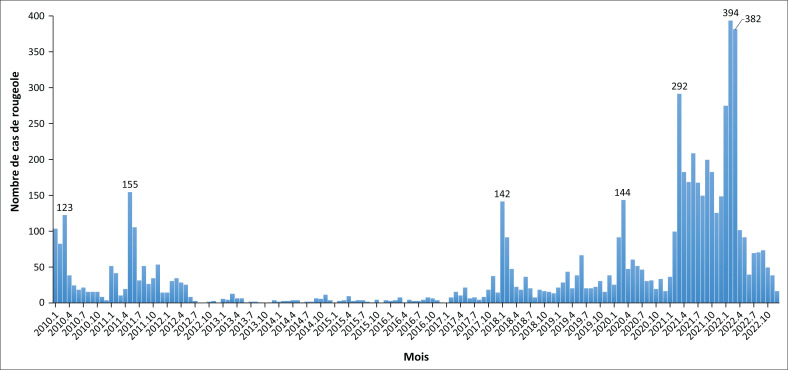
Courbe épidémiologique mensuelle des cas confirmés de rougeole en Côte d’Ivoire, 2010–2022 (*N* = 6 584).

**FIGURE 2-A1:**
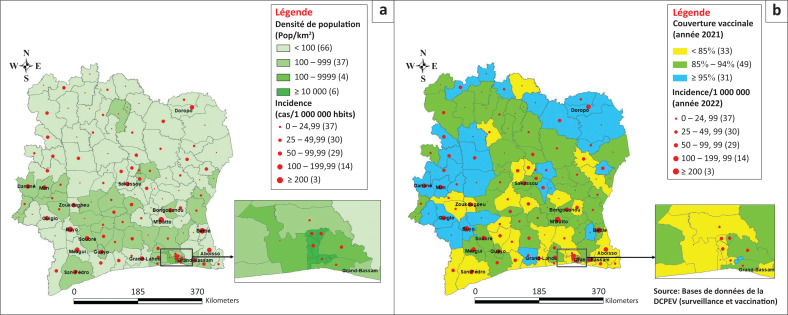
Répartition spatiale de l’incidence de la rougeole en 2022 selon la densité de la population et la couverture vaccinale administrative antirougeoleuse de 2021 dans les districts sanitaires, Côte d’Ivoire. (a) Densité de la population et incidence de la rougeole par district sanitaire en 2022 (*N* = 1606). (b) Couverture vaccinale en RR de 2021 et incidence de la rougeole par district sanitaire en 2022 (*N* = 1606).

**FIGURE 3-A1:**
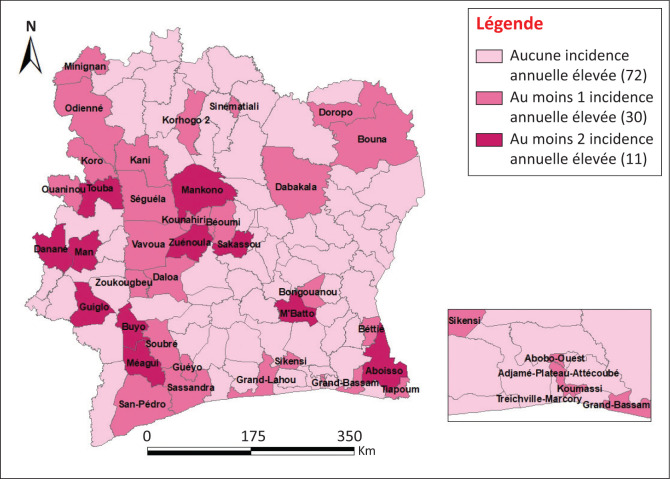
Répartition spatiale des districts sanitaires ayant au moins une incidence élevée de la rougeole, Côte d’Ivoire 2018–2022.

## Annexe 1

**TABLEAU 1-A1:** Analyse comparative des caractéristiques sociodémographiques et vaccinales des cas incidents dans les districts à incidence élevée avec une couverture vaccinale ≥ 95% et les districts à incidence élevée avec une couverture vaccinale < 95% en 2021.

Caractéristiques	Couverture vaccinale (≥ 95%, *N* = 263)				Couverture vaccinale (< 95%, *N* = 769)				Total, (*N* = 1032)				*p*-value
	*n*	%	Médiane	IIQ	*n*	%	Médiane	IIQ	*n*	%	Médiane	IIQ	
**Sexe** †	-	-	-	-	-	-	-	-	-	-	-	-	0.500
F	118	44.9	-	-	364	47.3	-	-	482	46.7	-	-	-
M	145	55.1	-	-	405	52.7	-	-	550	53.3	-	-	-
**Age**‡ (ans)	-	-	4	2–8	-	-	3	1–6	-	-	3	1–6	< 0.001
**Classe d’âge**	-	-	-	-	-	-	-	-	-	-	-	-	< 0.001
[0–5 ans]	161	61.2	-	-	564	73.3	-	-	725	70.3	-	-	-
[5–14 ans]	75	28.5	-	-	148	19.2	-	-	223	21.6	-	-	-
≥ 15 ans	27	10.3	-	-	57	7.4	-	-	84	8.1	-	-	-
**Statut vaccinal RR**	-	-	-	-	-	-	-	-	-	-	-	-	< 0.001
Non vacciné	204	77.6	-	-	682	88.7	-	-	886	85.9	-	-	-
1 dose	55	20.9	-	-	85	11.1	-	-	140	13.6	-	-	-
2 et plus	4	1.5	-	-	2	0.3	-	-	6	0.6	-	-	-

IIQ, intervalle interquartile.

† , Effectif (pourcentage);

‡ , Médiane (Intervalle interquartile).
